# Demographics, Clinical Characteristics, and Well-Being of Veterans with TBI and Dementia and Their Caregivers

**DOI:** 10.3390/geriatrics9050130

**Published:** 2024-10-08

**Authors:** Linda O. Nichols, Jennifer Martindale-Adams, Ronald T. Seel, Jeffrey K. Zuber, Paul B. Perrin

**Affiliations:** 1 Department of Preventive Medicine, University of Tennessee Health Science Center, Memphis, TN 38163, USA; linda.nichols@va.gov (L.O.N.); jmartindale@uthsc.edu (J.M.-A.); jeff.zuber@va.gov (J.K.Z.); 2 Caregiver Center, Lt. Col. Luke Weathers, Jr. Veterans Affairs Medical Center, Memphis, TN 38105, USA; 3 Department of Physical Medicine and Rehabilitation, School of Medicine, Center for Rehabilitation Science and Engineering, Virginia Commonwealth University, Richmond, VA 23298, USA; ronald.seel@vcuhealth.org; 4 School of Data Science and Department of Psychology, University of Virginia, Charlottesville, VA 22904, USA; 5 Central Virginia Veterans Affairs Health Care System, Richmond, VA 23249, USA

**Keywords:** traumatic brain injury, dementia, caregivers, veterans

## Abstract

Background: This study provides a detailed examination of older Veterans with traumatic brain injury (TBI) and dementia and their caregivers, focusing on Veterans’ demographic, clinical, functional, safety risk, and behavioral characteristics and caregivers’ demographic, clinical, and care-related characteristics and well-being. Methods: Veterans’ caregivers (N = 110) completed a telephone-based survey. Results: Veterans averaged eight comorbid health conditions, with over 60% having chronic pain, hypertension, post-traumatic stress disorder, or depression. Caregivers reported helping with an average of three activities of daily living, with the highest percentages of Veterans needing assistance with grooming, dressing, and bathing. Almost all Veterans needed assistance with shopping, cooking, medication management, housework, laundry, driving, and finances. Veterans averaged two safety risks, the most common being access to dangerous objects, access to a gun, and not being able to respond to emergency situations. Although Veterans averaged 14 behavioral concerns, caregivers reported that their family needs relating to TBI were generally met or partly met, and they voiced confidence in their ability to respond to behaviors and control their upsetting thoughts. Caregivers’ mean burden score was severe, while mean depression and anxiety scores were mild. Caregivers reported an average of 10.5 h per day providing care and 20.1 h per day on duty. Conclusions: The findings demonstrate the increased presence of impairments, safety risks, and behavioral issues in Veterans with comorbid TBI and dementia, as well as increased impacts on families’ burdens and care provision requirements. Clinicians should be alert for and educate TBI patients and caregivers on the warning signs of post-TBI dementia and its associated functional, behavioral, and safety risk profile, as well as challenges related to caregiver well-being. Healthcare policymakers must consider the increased caregiver demands associated with comorbid TBI and dementia, as well as the need for expanded long-term support and services.

## 1. Introduction

Traumatic brain injury (TBI) is a major public health problem in the U.S. and for military populations—the U.S. Department of Defense reported almost 500,000 TBIs among U.S. Service Members between 2000 and 2022 [1]. Veterans and Service Members (V/SMs) are at increased risk of TBI; major causes include blast injury, training, motor vehicle accidents, falls, and sports [2,3]. The prevalence of TBI has been shown to be higher in V/SMs compared to civilians [4]. TBI is a known risk factor for dementia [5,6,7], and studies have found that TBI increases the risk of dementia among veterans [8,9,10,11,12,13].

Individuals with post-TBI dementia have a somewhat different symptom profile than those with Alzheimer’s dementia (AD) [14,15,16,17,18]. People with post-TBI dementia have more depressive, agitated, and irritable symptoms [14,18]; greater gait dysfunction, motor slowness, and numbers of falls [14,18]; and greater levels of disinhibition [17] than people with AD alone. There is also evidence that individuals with post-TBI dementia have an increased risk of developing co-occurring medical and mental health conditions [19,20,21]. Common co-occurring medical conditions include cardiovascular or cerebrovascular disease [14,22,23]. Common co-occurring mental health conditions include depression [14,23,24,25,26,27], mixed affective disorders or bipolar disorder [26], and post-traumatic stress disorder (PTSD) [23,27]. People with post-TBI dementia are likely taking more medications [14] and require more hospital days and medical visits compared with non-TBI or non-dementia cohorts [28].

The research literature presents converging evidence on the diagnostic characteristics of individuals with post-TBI dementia, particularly in studies that leverage aggregate databases. Conversely, there is limited evidence about their demographic, behavioral and safety characteristics. In addition, there are few studies on the caregivers of individuals with TBI and dementia, including who they are and the care challenges they face.

Both the intervention for the randomized clinical trial (RCT) and the selection of measures was informed by the stress health process model, which conceptualizes the factors influencing whether caregivers experience stress and burden. Caregivers experience stress if they perceive that the demands placed on them are greater than their resources and their capacity to manage demands [29]. Challenges and demands can be related to the demographic (e.g., older age), clinical (e.g., health conditions), functional (e.g., need for assistance), or behavioral (e.g., distressing, or difficult to manage) concerns of the person who is being cared for. Environmental demands for the caregiver can be demographic (e.g., financial insecurity) or clinical (e.g., poor health). Caregivers can have resources to manage demands, both internal (e.g., perception of self-efficacy) and external (e.g., adequate support, few unmet needs). If they do not have resources, stress or physical, emotional, and cognitive responses (e.g., depression, anxiety, burden) can occur.

This study is a secondary analysis of a prospectively collected RCT registry related to caregiving for Veterans with TBI and dementia. As part of the RCT, a more in-depth measurement of Veterans with TBI and dementia and their caregivers was undertaken. For Veterans, demographic, clinical, functional, safety risk, and behavioral characteristics were examined. For their caregivers, demographic, clinical, well-being, and care-focused variables were analyzed.

## 2. Materials and Methods

### 2.1. Overview

The study of 110 caregivers of Veterans with TBI and dementia, “Supporting Caregivers of Veterans with TBI and Mixed Dementia: The REACH Hope Behavioral Intervention”, was sponsored by the U.S. Department of Defense (W81XWH2010756) from 15 August 2020–14 February 2024. The project was overseen by the Lt. Col. Luke Weathers, Jr. Veterans Affairs Medical Center at Memphis and Virginia Commonwealth University Institutional Review Boards (IRB) and the U.S. Army Medical Research and Development Command (USAMRDC), Office of Human and Animal Research Oversight (OHARO), and Office of Human Research Oversight (OHRO).

Veterans with TBI and AD diagnoses that could be identified by International Classification of Diseases (ICD) -9 or -10 codes, dependent on the date of diagnosis, using their electronic medical record (See Table 1) in the VA Health Care System, were identified through VA’s Corporate Data Warehouse. The household of each Veteran with an outpatient visit in the last 6 months (Veterans still living in the community) was sent a letter targeted to the Veteran’s caregiver, requesting the caregiver contact the research team if interested. Caregivers were eligible if they were the main caregiver, provided 4+ hours of care per day for at least 6 months, and endorsed a high burden on the Zarit Burden Inventory-4 [30,31]. One caregiver was enrolled for each Veteran.

### 2.2. Data

After caregiver enrollment and consent, baseline data were collected from the caregiver by telephone by a trained, certified research associate. Demographics from the Federal Interagency Traumatic Brain Injury Research Demographics Form [32], plus other variables of interest, were collected for the caregiver and Veteran: name; age; gender; race/ethnicity; marital status; relationship status; employment status; and income/month.

The presence (1) or absence (0) of comorbid health condition diagnoses for both were collected using the federally funded TBI Model Systems (TBIMS) Health Conditions List of 25 common diagnoses [33]. Conditions sorted using ICD-10 letter code structure. Medical- related conditions are classified using letters E, G, H, I, J, M). Mental- and behavioral disorder- related are classified as F. The Health and Health Services Use battery from the 2016 National Health Interview Survey (NHIS) assessed perceived health and health services use, including visiting healthcare providers, the emergency room, and the hospital [34]. These two measures have not been assessed for psychometric properties.

#### 2.2.1. Veteran Data

For Veterans, the branch of service, years served, and medical discharge were collected. Veteran function was assessed using the six-item Katz Activities of Daily Living (ADL) Scale [35] and the eight-item Lawton and Brody Instrumental Activities of Daily Living (IADL) Scale [36]. Each item was scored 0 (no help needed) or 1 (help needed). ADL and IADL items were summed separately, with higher scores indicating greater impairment. The ADL Cronbach’s alpha is 0.75 [37], and it is 0.84 for IADL [38].

Eight safety risks—access to dangerous objects, access to a gun, driving, responding to emergency situations, leaving the home and wandering, smoking concerns, aggression or violence, and harming self—were identified by project subject matter experts (SMEs), including clinicians, researchers, and caregivers. Higher scores indicate greater safety risk. Questions were either “Yes” (1) or “No (0).” Higher scores (0–8) reflect greater risk. The Cronbach’s alpha for these scores from the study data is 79.

To determine the presence of neuropsychiatric syndromes, the 12-item Neuropsychiatric Inventory (NPI) with an integrated caregiver distress scale was used [39,40]. The NPI has good test–retest correlation, r = 0.92 (*p* < 0.001, and intraclass correlation, r = 0.96 (*p* < 0.001) [40]. An additional 16 behaviors that are common in individuals with TBI and dementia were identified by the SMEs. The Cronbach’s alpha for all 28 items from the study data is 0.88. Scoring for all 28 items was present/absent during the past month, with the additional caregiver distress scoring on a scale from not at all (0) to very severe (5). The total distress rating for all 28 items ranged from 0 to 140, with the distress rating for the twelve NPI items being from 0 to 60.

#### 2.2.2. Caregiver Data

Burden, depression, anxiety, and frustrations captured caregiver well-being. Burden was measured with the Zarit Burden Interview (ZBI) short version. Its twelve items are scored 0 (never) to 4 (nearly always), with higher scores indicating more burden [30,41]. A summed score of >12 [42] or >17 [42] indicates high burden. The ZBI-12 has very good validity (rho = 0.95–0.97) and internal consistency (Cronbach’s alpha = 0.88 to 0.93) [42]. Depression items from the Patient Health Questionnaire (PHQ-9) [43] items, which were developed using the DSM-IV depression diagnostic criteria—the most updated version available at the time of measure development—are scored from 0 (not at all) to 3 (nearly every day). Scores are summed to characterize depression as minimal (0 to 4), mild (5 to 9), moderate (10 to 14), moderately severe (15 to 19), or high/severe (20 to 27). On the PHQ-9, major depressive disorder is suggested if five or more items, or the first two items, (interest and feeling depressed, also known as the PHQ-2) are scored positive (at least “more than half the days”). The PHQ-9 Cronbach’s alpha is 0.86 [43].

Anxiety was measured with the Generalized Anxiety Disorder scale (GAD-7), which focuses primarily on generalized anxiety disorder symptoms but also has good performance in detecting other anxiety disorders (panic disorder, social anxiety disorder, an PTSD) [44]. Item scoring ranges from 0 (not at all) to 3 (more than half the days) for an overall score of 0 to 21; higher scores indicate more anxiety. Clinical characterizations of anxiety are score of 0–4 (none), 5–9 (mild), 10–14 (moderate), and 15–21 (severe). Sensitivity is 0.89 and specificity is 0.82 [45]. Two items assessed caregiver frustration (e.g., feel like yelling at or hitting patient within past three months). Items are scored from never (0) to often (2). Higher scores indicate more frustration [46]. They are part of a larger caregiver Risk Appraisal Measure, which has a Cronbach’s alpha of 0.65 [47].

Care variables included length of caregiving, when the caregiver began care, and caregiving time spent on duty and in care activities per day, assessed with two questions from the Caregiver Vigilance Scale. The Cronbach’s alpha for the four-item scale is 0.66 [48].

The 37 items from the Family Needs Questionnaire (FNQ) [49,50] determine whether families have needs that have not been met. Items are scaled as follows: (1) met, (2) partly met, (3) not met, or not applicable. There are six subscales: (1) need for health information; (2) need for emotional support; (3) need for instrumental support; (4) need for professional support; (5) need for a community support network; and (6) need for involvement with care. Alpha reliability coefficients for the six subscales range from 0.78 to 0.89 [51].

Self-efficacy was measured with the Caregiving Self-Efficacy Scale-Revised [52,53], which contains fifteen items and three subscales: self-efficacy for obtaining respite (SE-OR), self-efficacy for responding to disturbing behaviors (SE-RDB), and self-efficacy for controlling upsetting thoughts (SE-CUT). Participants rate their level of confidence to perform each item on a scale from 0 (cannot do at all) to 100 (certain can do) (10-point increments). Subscale scores are reported separately. The Cronbach’s alphas for all three subscales are greater than 0.80 [53].

### 2.3. Analysis

Data were entered into Microsoft Access with logic checks, including ID validation, range and type verification, required field verification, parent–child field dependencies, and duplicate detection. Descriptive statistics were computed using R software. Means and standard deviation were calculated for continuous variables, and counts and proportions were calculated for categorical variables. Overall, 4% of data were missing.

## 3. Results

### 3.1. Veterans

#### 3.1.1. Demographics

The most common TBI diagnosis (64.5%) was ICD-9, 850–854: intracranial injury, excluding those with skull fracture (Table 1). The most common ICD-9 dementia diagnosis was 294.8: other persistent mental disorders due to conditions classified elsewhere, (35.5%), while the most common ICD-10 dementia diagnosis was F03.90–F03.91: unspecified dementia (25.5%).

**Table 1:** TBI and dementia International Classification of Diseases ICD-9 and ICD-10 codes used for data pull ^1^.

Diagnosis ICD Revision	Description	Codes	% of Sample
TBI			
ICD-9	Post-concussion syndrome	310.2	5.5
	Fracture of skull	800–804	4.5
	Intracranial injury, excluding those with skull fracture	850–854	64.5
	Other and unspecified open wound of head	873.8, 873.9	1.8
	Late effect of fracture of skull and face bones	905.0	0.9
	Late effect of intracranial injury without mention of skull fracture	907.0	8.2
	Other and unspecified injury to head face and neck	959.01	9.1
ICD-10	Post-concussional syndrome	F07.81	1.8
	Injuries to the head	S02.0, S02.1, S02.8, S02.9	0
		S06.0-S06.4, S06.8, S06.9	1.8
		S07.1	0
Dementia			
ICD-9	Dementia Other persistent mental disorders due to conditions classified elsewhere	290.0, 290.2–290.4	1.8
		294.8	35.5
	Alzheimer’s disease	331.0	4.5
	Senile degeneration of brain	331.2	0
ICD-10	Vascular dementia	F01.50–F01.51	8.2
	Dementia in other diseases classified elsewhere	F02.80–F02.81	18.2
	Unspecified dementia	F03.90–F03.91	25.5
	Alzheimer’s disease	G30.0, G30.1, G30.9	4.5

^1.^ A few Veterans had different codes than above for one of their two diagnoses. The code for Lewy Body Dementia (G31.83) was inadvertently included in early data pulls and *n* = 2 Veterans were enrolled. Another two were referred to study but had different TBI diagnoses: 310.9 Organic Brain Syndrome (*n* = 1) and S06.5 Traumatic Subdural Hemorrhage (*n* = 1).

The Veterans were mostly male (95.5%) with average age of 69.1 years (Table 2). Over ¾ (76.4%) were White and almost all were non-Hispanic (95.5%). Most (77.3%) were married. Seventy percent had some college or vocational education, although only 11.8% had graduated from college. Few (5.4%) were employed full- or part-time; half (50.9%) were disabled and 42.7% were retired. Slightly less than a third (29.1%) had been medically discharged from the military, and the average years of service was eight. About half (52.7%) were Army Veterans.

#### 3.1.2. Clinical Profile

Veterans averaged low healthcare use in the past three months, with an average of fewer than one visit to the emergency department, with number of visits ranging from 0 to 15 (Table 3). Veterans also averaged less than one hospital visit, with a range of 0 to 10 visits and 7.2 average days in hospital with a range of 0 to 40 days. The total number of comorbid health conditions reported was eight (8.15). Over 60% of Veterans had chronic pain, hypertension, post-traumatic stress disorder, and depression, and more than 50% had anxiety or high cholesterol. Fewer than 10% of Veterans reported liver disease, drug addiction, and Parkinson’s disease.

#### 3.1.3. Function

Caregivers reported helping with about three ADLs (2.87), with the highest percentages of Veterans needing assistance with grooming (61.8%), dressing (59.1%), and bathing (54.5%) (Table 4). Caregivers reported helping with slightly more than seven IADLS (7.06). Almost all Veterans needed assistance with shopping, cooking, medication management, housework, laundry, driving and finances.

#### 3.1.4. Safety Risks

Veterans had, on average, about two (1.82) safety risks (Table 4). The three most common were access to dangerous objects, access to a gun, and cannot respond to emergency situations, with over 50% of caregivers endorsing each. The two least-reported safety risks were concerns about harming self (10.0%) and smoking (7.3%).

#### 3.1.5. Behavioral Concerns

Caregivers reported that Veterans had, on average, 14.3 behavioral concerns (Table 5). For the twelve NPI items, on average, half were reported as occurring in the past month (6.3). The most common behavior reported was trouble remembering recent events, present in 90.9% of Veterans. Five others were reported for about ¾ of Veterans: losing or misplacing things, difficulty concentrating on a task, awakening during night, rising too early, or excessive naps, trouble remembering significant past events, and asking the same question over and over. The average distress rating for the entire 28 items was 44.8, with average distress ratings for the NPI items of 20.2.

### 3.2. Caregivers

#### 3.2.1. Demographics

The 110 caregivers were predominantly the wives of the Veterans (Table 2). Their average age was 65.1 years, and almost ¾ were White. Over 60% had some post-high school education. Slightly over 20% of participants were employed full- or part-time, and most (78.1%) did not find it difficult to pay for basics. Participants had been providing care, on average, 14.1 years. Most (56.4%) became caregivers when the Veteran was no longer able to care for themselves, with an additional 21.8% beginning care when the Veteran returned injured. Almost a third (32.7%) reported they had a condition that made caregiving difficult.

Caregivers’ average scores suggested that their family needs relating to TBI were generally met or partly met. They were more confident in their ability to respond to behaviors and control their upsetting thoughts than in their ability to obtain respite.

#### 3.2.2. Clinical Profile

The mean score for burden was above the severe cut point (>17), while mean depression and anxiety scores were consistent with mild symptoms (<10) (Table 2). Caregivers reported, on average, moderate stress. Almost 90% reported they felt like screaming/yelling at their loved one, and almost 10% reported having to keep themselves from hitting the Veteran. Caregivers reported, on average, 10.5 h per day providing care and 20.1 h per day on duty.

Most caregivers (60.9%) reported that their health status was about the same as a year ago, although 26.4% reported worse health. Caregivers reported about five chronic conditions, with around half reporting hypertension and high cholesterol. Almost ¾ had seen their healthcare provider in the past three months, and emergency room and hospital use were low (Table 3).

## 4. Discussion

Similar to Veterans with comorbid TBI and dementia described in other studies [18,19], this sample of Veterans with post-TBI dementia was predominantly male, White, and non-Hispanic. Similar to large-scale examinations of Veterans with TBI and dementia, common comorbid conditions were hypertension or cardiovascular diagnoses [50]. Chronic pain was more common in this sample. Psychiatric comorbidities were similar, including post-traumatic stress disorder, depression, and anxiety [54]. With an average age of 69.1 years, our sample was younger than that reported in one study (78.6 years) [55] but similar in age (66.9 years) and education level to that found in another small study [18].

Our sample may provide clues about the broader population of Veterans with TBI and dementia. Older Veterans with TBI and dementia and their caregivers are not just managing two conditions. One finding that has been seen in other studies of Veterans with TBI and dementia was the large number of comorbid conditions [19,56]. Veterans had, on average, more than eight comorbidities, most of which involve extensive symptomatology and precise medication management. These chronic conditions are coupled with the behavioral concerns identified by their caregivers, largely involving memory deficits, and their limited ability to independently manage daily activities of living and instrumental activities of daily living, particularly those requiring executive function. All these clinical factors identify a vulnerable population, requiring extensive care and supervision to enable them to remain at home.

On average, the caregivers of these Veterans with both TBI and dementia had been providing care for 14.1 years. They were predominantly older wives, with an average age of 65 years and some post-high school education. Most were not working. Consistent with the care of a vulnerable population, caregivers spent an average of 10.3 h per day in care activities and 20.1 h per day on duty. These figures are similar to those of caregivers of Veterans with dementia only (10.9 h in care and 20.4 h on duty) [57]. They were burdened, with mean scores above the severe cut point, and 90% reporting they felt like yelling at their loved one during the past three months. Interestingly, these caregivers had mild depression and anxiety, similar to caregivers of Veterans with dementia only [57]. Caregivers reported an average of 14 behaviors occurring for the Veteran during the past month. Two of the least-reported behaviors—wandering and inappropriate sexual behavior—were the most distressing.

Other studies have recommended increased clinical attention to identify individuals with TBI who may develop dementia. It is important for primary providers to monitor the neurocognitive trajectories of Veterans with TBI to facilitate the timely detection of neurodegeneration. Early detection may prompt treatments that can slow cognitive decline and education that may foster more effective care transitions. When individuals with comorbid TBI and dementia have been identified, special care should be given to determine the level of disability that is present, both from the symptoms associated with TBI and dementia and from symptoms and challenges associated with other conditions. The findings from the current study also support the administration of measures of functional independence to assess Veterans’ functional level and promote effective rehabilitation efforts.

Our study findings suggest other actions for healthcare policymakers and clinicians who are providing care to individuals with TBI and their caregivers. High rates of dementia risk factors, such as hypertension/high cholesterol/cardiovascular disease, diabetes, post-traumatic stress disorder, depression, and sleep disorders, coupled with persons with a TBI-diminished cognitive capacity to self-manage these conditions, creates a perfect storm for developing dementia. Early healthy lifestyle interventions and the proactive management of chronic health conditions that mitigate associated dementia risk are critical to delivering quality clinical care to Veterans with TBI. For those with comorbid TBI and dementia, managing ADL and IADL needs as well as distressing behaviors may be of greater daily concern for their caregivers than the high rates of comorbid conditions and medication needs [14,22,23]. The behaviors and safety risks for these Veterans were similar to those of Veterans with dementia, suggesting that even long-time caregivers of an individual with TBI may have new challenges with the addition of dementia. In addition, caregivers’ clinicians may need to be more alert to burden levels and hours of care, in addition to depression and anxiety, which are more likely to be screened for.

This study highlights the importance of creating supportive community and healthcare systems for aging Veterans and their caregivers. In addition to training for clinicians, for example, adult day cares could accommodate post-TBI dementia with additional staff training on TBI. Training for friends, families, and neighbors could demystify the condition and help identify areas where families need assistance and how to provide it. Community training for police and first responders could help alert them to Veterans who may still be active in the community or neighborhood.

The present study has several limitations that reflect opportunities for future research. The sample size of 110 Veterans is rather small, and the majority of the sample were White and male, limiting the generalizability of the findings for female Veterans, racial/ethnic minority Veterans, and civilians. Future studies may consider using a larger sample and recruiting from underrepresented groups. We relied on caregiver reports to gather demographic and clinical characteristics of the Veterans, which could be prone to errors and bias. Additionally, a limitation associated with using aggregated data is that the sample selection used clinical diagnoses in VA medical records, which were not assigned using a consensus definition for TBI or dementia. We did not collect data on TBI severity, timing of dementia onset, mechanism of injury, or whether the brain injury was related to military service, all of which may be variables of interest for future research to focus on.

## 5. Conclusions

This article describes the demographic, clinical, functional, safety risk, and behavioral characteristics of a group of older Veterans with post-TBI dementia and their caregivers. The findings increase our knowledge of the functional level and patterns of neurobehavioral impairments in Veterans with TBI and dementia and the well-being of their caregivers. The results demonstrate the increased presence of impairments, safety risks and behavioral issues in Veterans with comorbid TBI and dementia, and the increased impacts on families’ burdens and care provision requirements. Clinicians should be alert for and educate TBI patients and caregivers on the warning signs of post-TBI dementia and its associated functional, behavioral, and safety risk profile. Healthcare policymakers must consider the increased caregiver demands associated with comorbid TBI and dementia, as well as the need for expanded long-term supports and services.

## Figures and Tables

**Table 2:** Veterans’ and caregivers’ demographics (N = 110).

Variable	Veteran Mean (SD) or %	Caregiver Mean (SD) or %
Age, years	69.1 (11.4)	65.1 (10.7)
Male	95.5	92.7
Hispanic	4.5	6.4
Race		
American Indian/Alaskan Native	-	0.9
Asian	0.9	1.8
Black/African American	19.1	18.2
Native Hawaiian/Pacific Islander	0.9	0.9
White/Caucasian	76.4	72.7
Other	0.9	2.7
More than one race	0.9	1.8
Marital Status		
Divorced	12.7	10.0
Married/Living as married	77.3	82.7
Single	6.4	1.8
Widowed	3.6	5.5
Education		
Junior/high school	2.7	3.6
High school graduate/GED	27.3	16.4
Some college/associate degree	41.8	34.5
Vocational/training school	6.4	6.4
College graduate	11.8	20.0
Employment Status		
Not currently employed		
Disabled/not employed	51.8	
Retired	42.7	77.3
Paid employment		
Full-time	3.6	11.8
Part-time	1.8	10.0
Service Branch		
Air Force	13.6	
Army	52.7	
Marines	20.0	
Navy	11.8	
Coast Guard	0.9	
Years of service	7.8 (8.8)	
Medically discharged	29.1	
Relationship to Veteran		
Wife		76.4
Partner		0.9
Mother		4.5
Brother		3.6
Sister		3.6
Daughter		3.6
Son/step-son		1.8
Gross monthly income, USD		5330 (7620)
Hard to pay for basics		
Not at all difficult		54.5
Not very difficult		23.6
Somewhat difficult		20.9
Very difficult		0.9
Time providing care, years		14.1 (13.1)
Began care		
as soon as Veteran returned injured		21.8
Veteran no longer caring for self		56.4
Previous caregiver no longer caring for Veteran		11.8
Other		10.0
Family needs, (FNQ), (1–3)		1.8 (0.4)
Health Info (1–3)		1.5 (0.5)
Emotional Support (1–3)		2.2 (0.5)
Instrumental Support (1–3)		2.1 (0.6)
Professional Support (1–3)		2.0 (0.7)
Community Support Network (1–3)		1.4 (0.5)
Involvement with Care (1–3)		1.4 (0.6)
Caregiving Self-Efficacy, (0–100)		66.4 (18.8)
Obtaining Respite (0–100)		47.5 (29.9)
Responding to Behaviors (0–100)		77.0 (23.1)
Controlling Upsetting Thoughts (0–100)		73.2 (21.6)

**Table 3:** Veterans’ and caregivers’ clinical characteristics (N = 110).

Variable	Veteran Mean (SD) or %	Caregiver Mean (SD) or %
Health services use		
ER visits, past 3 months	0.77 (2.0)	0.1 (0.3)
Hospital visits, past 3 months	0.29 (1.1)	0.0 (0.2)
Days in hospital, past 3 months	7.1 (10.7)	4.7 (1.5)
Total number health conditions (0–25)	8.12 (3.9)	4.8 (3.0)
Reported health conditions		
Medical-related		
Cataracts	48.2	34.5
Chronic pain	66.4	32.7
Congestive heart failure	18.2	2.7
Diabetes, high blood sugar, sugar in urine	35.5	24.5
Emphysema or asthma or COPD	26.4	18.2
Heart arrhythmias	18.2	14.5
High blood cholesterol	56.4	51.8
Hypertension or high blood pressure	63.6	54.5
Liver disease	10.0	3.6
Myocardial infarction or heart attack	15.5	4.5
Osteoarthritis	37.3	38.2
Parkinson’s disease	7.3	.9
Pneumonia	30.9	23.6
Rheumatoid arthritis	12.7	14.6
Sleep disorder	49.1	22.7
Stroke	34.5	7.3
Mental- and behavioral disorder-related		
Alcoholism	18.2	5.4
Anxiety	57.3	40.0
Attention deficit disorder/attention deficit hyperactivity disorder	17.3	5.4
Bipolar disorder/manic-depression	20.9	6.4
Depression	61.8	40.9
Drug addiction	9.1	1.8
Obsessive-compulsive disorder	18.2	2.7
Panic attacks	19.1	17.2
PTSD (post-traumatic stress disorder)	62.7	9.1

**Table 4:** Veterans with TBI and dementia functional characteristics and safety risks (N = 110).

Variable	Mean (SD) or %
Total ADL (0–6)	2.87 (2.09)
Bathing	54.5
Dressing	59.1
Grooming	61.8
Toileting	38.2
Transferring	34.5
Eating	39.1
Total IADL (0–8)	7.06 (1.28)
Shopping	94.5
Cooking	95.5
Managing medications	93.6
Doing housework	92.7
Doing laundry	94.5
Driving or using public transportation	89.1
Managing finances	95.5
Using the phone	50.9
Total safety risks (0–8)	1.82 (1.31)
Access to dangerous objects	58.2%
Access to gun	51.8
Cannot respond to emergency situations	50.9
Aggressive/violent to others or property	39.1
Leave home and wander outside	31.8
Driving	27.3
Concerns about harming self	10.0
Smoking concerns	7.3

**Table 5:** Veterans with TBI and dementia behavioral concerns (N = 110).

Variable	Present Past Month Mean (SD) or %	Distress Level ^1^ Mean (SD) or %
Total behavioral concerns (0–28)	14.3 (4.8)	44.8 (24.3)
Total NPI concerns (0–12)	6.3 (2.7)	20.2 (11.7)
Behavioral concerns		
Trouble remembering recent events	90.9	3.0 (1.6)
Lose or misplace things	79.1	3.2 (1.4)
Difficulty concentrating on a task	79.1	3.1 (1.4)
Trouble remembering significant past events	76.4	2.6 (1.6)
Ask the same question over and over	74.5	3.0 (1.6)
Difficulty communicating (forgetting/misusing words)	68.2	3.0 (1.5)
Start, but not finish, things	60.9	3.0 (1.5)
Accidents of the bowel or bladder	57.3	3.0 (1.8)
Disorientation/confusion about surroundings	42.7	2.9 (1.3)
Have problems with bathing; refusing to bathe	37.3	3.6 (1.5)
Aggressive to others verbally	36.4	3.8 (1.3)
Have problems with dressing; refusing to get dressed	31.8	2.9 (1.4)
Get upset when friends or family visit	20.0	3.4 (1.3)
Inappropriate sexual behavior	16.4	4.2 (0.9)
Wandering outside the home	16.4	3.9 (1.2)
Misuse alcohol or other substances	9.1	3.4 (1.4)
Neuropsychiatric Inventory (NPI) concerns		
Awaken during night, rise too early, or excessive naps	77.3	3.3 (1.4)
Impatient and cranky	65.5	3.2 (1.3)
Resistive to help, hard to handle	63.6	3.4 (1.2)
Seem sad, depressed	60.9	3.5 (1.1)
Become upset when separated from you, signs of nervousness	60.0	3.4 (1.4)
Seem less interested in usual activities	56.4	3.2 (1.4)
Seem to act impulsively	51.8	3.1 (1.5)
Engage in repetitive activities	50.9	3.0 (1.5)
Lost or gained weight, or had a change in food	44.5	2.9 (1.7)
Hallucinations such as false visions or voices	41.8	2.8 (1.5)
Have false beliefs	37.3	3.4 (1.3)
Appear to feel too good or act excessively happy	19.1	2.4 (1.4)

^1.^ Caregiver distress scored not at all (0) to very severe (5) for each item occurring in past month. Total distress rating for all 28 items ranged from 0 to 140 and 0 to 60 rating for twelve NPI items.

## Data Availability

Data are available upon request. Data are in the Caregiver Center repository at the Lt. Col. Luke Weathers, Jr. Veterans Affairs Medical Center at Memphis.
